# Validation of a Flow Cytometry Based Binding Assay for Evaluation of Monoclonal Antibody Recognizing EGF Receptor

**DOI:** 10.3797/scipharm.1104-18

**Published:** 2011-07-03

**Authors:** Mercedes Cedeño-Arias, Javier Sánchez-Ramírez, Rancés Blanco-Santana, Enrique Rengifo-Calzado

**Affiliations:** Center of Molecular Immunology, 216 St and 15 Ave. Atabey, Playa. POBox 16040, Havana 11600, Cuba

**Keywords:** Binding Assays, Method validation, Monoclonal antibody, Nimotuzumab

## Abstract

An ideal test used to characterize a product must be appropriate for the measurement of product quality, manufacturing consistency, product stability, and comparability studies. Flow cytometry has been successfully applied to the examination of antibodies and receptors on membrane surfaces; however, to date, the analytical validation of cytometry based assays is limited. Here we report on the validation of a flow cytometry-based assay used in the evaluation of nimotuzumab binding to cells over-expressing EGFR on cell surface. The assay was validated by examining, assay robustness, specificity, repeatability and intermediate precision. The assay was highly specific, robust for all studied factors except for cell fixation with 1% paraformaldehyde and met criteria for precision with RSD < 2%. In addition the assay has stability-indicating properties evidenced by the ability to detect changes in mAb degraded samples. Most importantly, the assay demonstrated to be useful for its intended use.

## Introduction

Most biological products act through some form of binding to another moiety. Fluorescence flow cytometry is used in the observations and analysis of the interaction of fluorescently labeled ligands and their cellular receptors. Binding assay by flow cytometry is commonly used to characterize the activity of the product through binding to its specific receptor. When the mechanism of action of a monoclonal antibody (mAb) is to block the binding of ligand to cell surface receptor, in vitro binding assay can be used as surrogate potency test using the therapeutic mAb [1].

The development of accurate and well characterized assays for biological products is vital for their development as therapeutic drug [2]. The biological activity measured should be closely related to the product's intended biological effect and ideally it should be related to expected clinical response [3, 4].

Nimotuzumab (also known as h-R3) is an IgG1 humanized anti–epidermal growth factor receptor (EGFR) mAb that was obtained by complementarity determining regions grafting of a murine mAb to a human framework [5]. Nimotuzumab binds to domain III of the extracellular region of the EGFR and interferes with EGF binding [5, 6]. At present, nimotuzumab is one of the very few anti-EGFR monoclonal antibodies that have been approved for therapeutic use in cancer treatment.

The appropriate validation of any bioassay used in the characterization of biological products is critical. Regulatory agencies provide general guidance on validation of analytical methods [3, 4] although these are not specific to biological assays. Validation of a cell-based bioassay [7], and immunoassays for bioanalysis has been reviewed [8], however very few information is available for validation of flow cytometry assays [9].

Here, we report on the validation study (assay robustness, specificity and precision) of the nimotuzumab binding assay by Flow Cytometry.

## Results and Discussion

The validation of analytical procedures is an important part in the registration application for a new drug [2]. Based on the method characteristics and requirements of the International Conference on Harmonization (ICH) guidelines, each analytical procedure must be validated with respect to parameters which are relevant to its performance [8, 10].

### Reagent titration

**Fig. 1.**
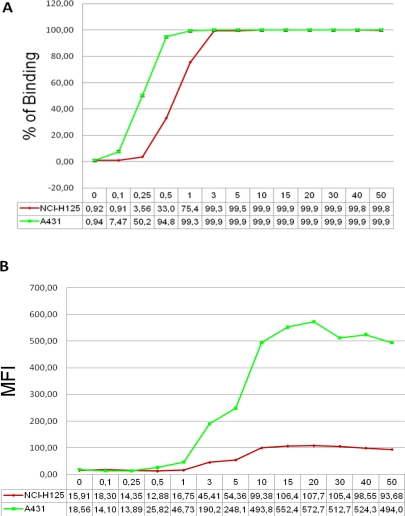
A typical dose-response curve for a FACS-binding assay data set is shown. Graph in (A) shows % of binding and in (B) mean of fluorescence intensity of nimotuzumab on cell surface EGFR in two different tumor cell lines.

Cytometry can measure both phenotypic and functional parameters from cells and has been used in the diagnosis and monitoring of progression of diseases and also to demonstrate biological activity of drugs [9]. In order to determine the optimum concentration of nimotuzumab used in the assay the reagent was titrated on two epithelial cell line over-expressing EGFR and a titration curve was created. A typical standard curve is shown in figure 1. For the data shown in figure 1a, saturation of binding was achieved at a concentration of 3–5 μg/mL of nimotuzumab when % of binding was reported. While mean of fluorescence intensity (MFI) was examined (Figure 1b), the saturating mAb concentration was of 10–20 μg/mL in both cell lines. As reported before, A 431 showed a higher antigen density [11] on cell surface than NCI-H125 cell line [12]. In the subsequent experiments we always used the parameter % of binding for the analysis because demonstrate less variability of the results when the assay is performed multiple times (RSD less than 1% at 3 μg/mL of mAb). However, when MFI was measured the inter-assay variability shown RSD higher than 10%.

### Assay robustness

Robustness testing is part of method validation [3, 13]. Especially in the pharmaceutical industry, extensive method validation is required in order to meet the regulations set by the regulatory agencies. For robustness study the factors selected have to reflect potential changes that may occur during validation process.

**Fig. 2.**
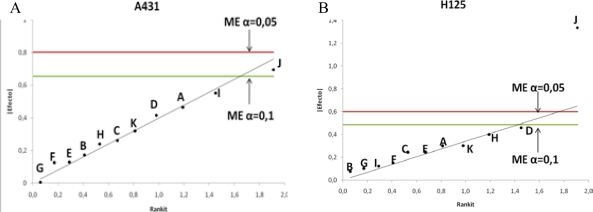
Half-normal probability plot for the effects estimated in Plackett-Burman design (from Table 2) with the identification of margin of error (ME). Variable: % of binding of nimotuzumab evaluated by flow cytometry on A 431 (A) and H125 (B) cell lines respectively.

The robustness of the assay was performed also on two cell lines. Ten factors were selected from the analytical procedure to be examined. As shown in table 1 in this study qualitative and quantitative factors were evaluated. The factors were investigated in a Plackett-Burman design and the levels for each factor used are given in tables 1 and 2. In each of the 12 experiments performed, the average from three replicates of % of binding is shown in table 1. The statistical analysis described above and the results are given in Table 2 and plotted in figure 2.

Only the cell fixation with 1% formaldehyde (factor J) represents a significant factor affecting the response in both cell lines at α=0.1. After formaldehyde fixation cells showed a higher % of mAb binding and in MFI probably due to gain in surface marker detection in a subset of cells (above background level) most evident in NCI-H125 cell (significant also at α=0.05). The results found with the statistical and graphical interpretation agreed.

Several investigators have used prefixed cells for analysis by flow cytometry. The most widely used compound is paraformaldehyde, generally in a concentration of 1 or 2% prior to antibody staining. Fixing samples with formaldehyde increases cell permeability and causes surface protein cross-linking that may alter staining of both intra- and extracellular markers [14]. The time of fixation varies from several minutes to overnight. Both over and under-fixation usually results in a loss of antigenicity. The reason for the increase in fluorescence, though, is not clearly understood. Studies that have examined fixation on sample preparation have produced contradictory results.

### Specificity

Assay specificity is an important and critical issue. The nonspecificity results from the interferences of compounds with similar physicochemical properties to those of the analyte and it is sometimes referred as the matrix effect [15]. The specificity of the assay was evaluated to assure mAb binding to antigen on cell surface. As we expected, low background binding was observed from samples with no mAb (< 5% for isotype T1hT irrelevant mAb and different mAb-free matrix solutions). While, nimotuzumab is bound specifically to cell surface (≥ 97 %) in the relevant samples containing mAb (reference standard and mAb in culture supernatant).

Although the high percentage of binding of degraded (pH adjusted to 10 and incubated at 40°C for 168 hours) sample of mAb was evidenced, the result was lower than with non-degraded molecule (Table 3).

**Fig. 3.**
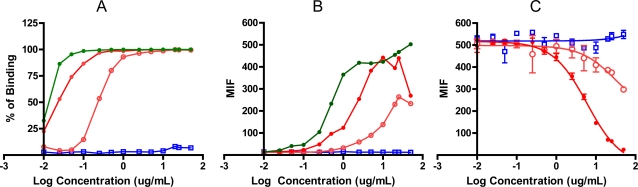
Binding of nimotuzumab to EGFR expressed on A431 cell line. The graphs represent a comparison between native and degraded-mAbs. [A], % of binding and [B], mean intensity of fluoresce of degraded isotype control ( 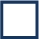), degraded nimotuzumab ( 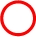), native nimotuzumab ( 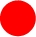), or native FITC-conjugated nimotuzumab ( 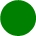), antihuman/FITC was used as secondary Ab when non-conjugated mAb was evaluated. [C], Results of competition binding assay, concentrations from 0.01 to 50 μg/mL of native and degraded mAbs were incubated for 30 min at 4°C in the presence of FITC-Conjugate nimotuzumab (2.5 μg/mL).

To evaluate the occupation of EGFR by degraded mAb, a competition assay was performed and the inhibition of binding of FITC-conjugated nimotuzumab exerted by the degraded isotype control, native and degraded nimotuzumab was measured (Fig. 3).

Native mAb showed a higher degree of receptor occupation than degraded mAb. In contrast degraded isotype negative control showed no bound to the receptor (Fig. 3A–B). Increasing concentration of native mAb may be more effective than degraded in displacement the labeled antibody (Fig. 3C). This result probably due to the native conformation of mAb was destroyed during degradation process with lost biological mAb activity or it is not sufficient to make action. To confirm that mAb was maximally degraded, the electrophoretic procedures (SDS-PAGE and isoelectric focusing) were used (data no shown). The concentrations at which 50% effect occurred were 6.01 and 30.7 μg/mL for native and degraded mAb respectively.

### Precision (repeatability/reproducibility)

Precision is a measure of the ability of the method to generate reproducible results [3, 4]. The precision of assay was evaluated using three separated determinations with two cell lines for repeatability (intra-assay) and intermediate precision (inter-day and inter-person variability). In order to calculate the assay precision, the average, SD and RSD were determined from the six replicates determined on individual days. Representative data is shown in table 4.

Intra assay precision expresses the precision under the same operating conditions (during a single analytical run). For intra assay precision, two analyst tested samples in three repeats for both NCI-H125 and A431 cell lines using nimotuzumab as culture supernatant or as purified mAb. Average % of binding (N=6 each) were found ≥ 98%.

RSD [(intra assay SD/Intra assay average calculated value) × 100] was < 2% which is considered acceptable.

Inter assay precision (Intermediate precision) is defined as the variability of a sample tested in multiple assays over than one day. The data are expressed as average value and RSD [(inter assay SD/Inter assay average calculated value) × 100]. Inter assay precision was also assessed by two analysts in three independent assays. Similarly, average (N=18) was also satisfactory with RSD < 2%. This degree of precision compares well to reported for binding assays with RSD between 5–20% is considered acceptable [16]. Usually acceptable repeatability of the results within one day and day-to-day was observed. The result obtained from inter-person reproducibility (N=36) is <1%, also indicated a good method precision. Assay precision for flow cytometry binding assay met the general criteria with RSD <20% (intra assay) and < 25% (inter assay) [8].

## Experimental

### Antibodies and sample preparations

Nimotuzumab (mAb-containing sample) as culture supernatant, the internal reference standard and other two purified lots were used. As mAb-free matrix sample, a batch of buffer solution used in drug formulation and complete cell culture media were used. T1hT (IgG1, anti CD6 mAb at 5 mg/mL) manufactured by Biocon, India was used as isotype control. Degraded sample of both, nimotuzumab and T1hT mAbs, were prepared by pH adjusted to 10 with 1M NaOH and incubated at 40°C for 168 hours). All mAbs were kept at 2–8°C until use.

All working dilutions of samples were obtained by diluting the stock solution (5.0 mg/mL) with FACSflow, first at 50.0 μg/mL and further diluting to final concentrations. All samples were prepared daily prior to assay.

In robustness assays reagents from two different brands were used, Gibco-BRL, USA and PAA, Austria: Dulbecco’s Modified Eagle Medium (DMEM) from Gibco (12800-017) and PAA (E15843). Fetal bovine serum (FBS) from Gibco (10082) and PAA (A 15-211). 0.25% Trypsin–EDTA from Gibco (25200-056) and PAA (L11-660). 1% formaldehyde (Sigma-Aldrich, USA) prepared in FACSflow solution (BD 342003, USA) was used for cell fixation.

### FITC conjugation of Nimotuzumab

Nimotuzumab was conjugated with reactive fluorescein isothiocyanate (FITC) according [17] with minor deviation. Briefly, 7.5 mg (1.5 mL) of mAb was exchange over a Hitrap Desalting column from General Electric (17-140801) in reaction buffer (500 mM carbonate, pH 9.5). Covalent conjugation was made wrapped in foil and incubated for 1.5 h with gentle rotation at room temperature. Unreacted FITC was removed and the antibody was exchanged into storage buffer (PBS, pH 7.4).

### Cell line and culture conditions

The human epidermoid carcinoma cell line A431 (CRL-1555) and non small lung cell carcinoma NCI-H125 (CRL-5801) were obtained from the American Type Culture Collection (ATCC, Rockville, MD). Cells were cultured in DMEM (Gibco, USA) supplemented with 5% and 10% respectively of FBS (Gibco, USA) under standard conditions (37°C in humidified atmosphere containing 5% CO2). Cells were used around 90% of confluence at time of assay, detached with 1 mL of Trypsin-EDTA for 10 min (A431) and 5 min (NCI-H125) respectively and then resuspended in complete growth medium. Cell concentration and viability were determined with a hemacytometer using Trypan-blue exclusion method.

### Surface binding mAb analysis

Cells were resuspended in complete culture medium and kept in a water bath at 37°C for 60 min. Cell concentration was adjusted at 1×10^6^ cells/mL and the staining was performed in 2.5 ×10^5^ cells. The mAbs were added according to the experimental plan, and cells were stained for 30 min at 2–8°C. The cells were washed with 2 mL of FACS flow and spin out at 246 g for 10 min at 4°C. FITC-conjugated rabbit anti human IgG (Dako F0056, Denmark, 1:60) was added and cells were stained for 30 min at 2–8°C. Cells were washed as described before and resuspended in 200 μL of FACSflow for flow cytometric analysis. In robustness analysis, cells were resuspended in 200 μL of 1% formaldehyde and incubated for 24 hours at 2–8°C before flow cytometric analysis. A Becton Dickinson FACSCan instrument using 488 argon lasers was used.

## Assay validation

### Robustness

Assay robustness was defined as how “reproducibly” the assay performed over time after deliberate manipulation of environmental parameters. A Plackett-Burman [13] design for 11 factors (N=12) was performed to investigate the influence of certain experimental an environmental factors on assay outcome (Table 5). For each factor, the nominal level was considered as in a regular assay (see in table 5 and described before in surface binding mAb analysis) and the extreme level (− or +) was considered as a level deviation from the nominal one. All experiments were performed in randomized order and carried out in short space of time in four experimental blocks including three experiments each: B-I (experiments 1, 5 and 11); B-II (experiments 2, 4 and 7); B-III (experiments 3, 6 and 9) and B-IV (experiments 8, 10 and 12).

### Specificity

The specificity of the assay is the ability of the assay to measure the analyte unequivocally in the presence of other components in the sample. Here we evaluated the matrix interference (excipients used in culture supernatant and in final buffer formulation), irrelevant mAb (isotype control), positive sample (nimotuzumab in culture supernatant and formulated as final product, including an internal references standard) and degraded sample.

### Precision (repeatability/reproducibility)

Assay precision was determined evaluating internal references standard, and nimotuzumab (in culture supernatant and purified as a final product) at 3 different times. Each run of the assay was performed on three separated occasions. Assay was assessed by six replicates of the drug over time within the same day (intra-assay repeatability) and on three different days (inter-day variation), and across individuals (inter-person reproducibility). In order to calculate the assay precision, the average, standard deviation (SD) and relative standard deviation (RSD) as [(SD/average) × 100] were determined.

### Stability

The suitability of FACS assay to be stability-indicative of nimotuzumab was determined by a competition binding immunoassay. Increasing concentrations of mAbs (native and degraded samples) were added to cell suspension in the presence of a constant amount (2.5 μg/mL) of FITC-conjugated native mAb. The assay was performed as described previously in surface binding mAb analysis. The % of binding and median of fluorescence intensity (MFI) curves generated from the results of duplicate were averaged. The 50% of response was also determined using GraphPad Prism program.

### Data analysis

After acquisition, all raw data were analyzed using WinMDI 2.8 (free Software http://facs.scripps.edu/software.html). Percent of nimotuzumab positive cells were obtained from upper right quadrant of cell gated in forward and side scatter profile. Median of fluorescence intensity (MFI) value was determined for positive population of cells.

In robustness assay, statistical and graphical analyses of the effects were performed for interpretation. Algorithm of Dong [13] was used to identify significant effects and a half-normal probability plots were drawn to indicate visually relevant effects. The average, SD, standard error and RSD were calculated using Excel 2007 (Microsoft).

### Overall criteria for evaluation

Percent of positive cells of isotype mAb (T1hT) and placebo solutions should be not more than 5% of positive cells of internal reference standard and nimotuzumab samples should be more than 97%. Percent of nimotuzumab degraded sample should be less than 97%. RSD less than 3% was considered an acceptable parameter.

## Conclusion

Development of a convenient, robust, specific and precise procedure is important in quality control laboratories. A simple and sensitive immunoassay has been developed for the determination of nimotuzumab binding activity by flow cytometry. Validation was performed in accordance with the ICH guidelines and under current good manufacturing practices (cGMP). These studies confirm that flow cytometry is a useful and expeditious technique for detecting mAb binding on cell surface receptors maintains low assay variation. The assay showed a high level of specificity and was found to be free of interference through the validation process. Also the assay has stability-indicating properties as evidenced by the ability to detect changes in binding of mAb degraded samples.

## Figures and Tables

**Tab. 1.** Plackett-Burman design for 11 factors. % of nimotuzumab binding in both A 431 and NCI-H125 cell lines.

	**Factor**											**% of Binding**	

**Exp.**	**A**	**B**	**C**	**D**	**E**	F*	**G**	**H**	**I**	**J**	**K**	**A431**	**H125**
1	+	+	−	+	+	+	−	−	−	+	−	99,73	99,73
2	−	+	+	−	+	+	+	−	−	−	+	98,73	97,80
3	+	−	+	+	−	+	+	+	−	−	−	99,23	98,70
4	−	+	−	+	+	−	+	+	+	−	−	99,23	99,47
5	−	−	+	−	+	+	−	+	+	+	−	99,73	99,93
6	−	−	−	+	−	+	+	−	+	+	+	99,83	100,00
7	+	−	−	−	+	−	+	+	−	+	+	99,57	99,70
8	+	+	−	−	−	+	−	+	+	−	+	99,60	98,13
9	+	+	+	−	−	−	+	−	+	+	−	99,87	99,37
10	−	+	+	+	−	−	−	+	−	+	+	99,83	100,00
11	+	−	+	+	+	−	−	−	+	−	+	99,87	98,20
12	−	−	−	−	−	−	−	−	−	−	−	97,73	98,43

Average												99,41	99,12
RSD												0,63	0,82

Exp…No of experiments;

+…High level;

−…Low level;

* …Dummy factor.

**Tab. 2.** Effects of factors on the response of % of binding of nimotuzumab on cells lines over-expressed EGFR measured by Flow cytometry.

	**Effects**	
**Factors**	**A 431**	**NCI-H125**
Cell Culture Media manufacturer	−0,005	0,103
Fetal Bovine Sera manufacturer	0,129	0,245
Time of Trypsinization	0,551	0,123
Trypsin-EDTA manufacturer	0,171	−0,077
Incubation time for EGFR recovery	0,465	−0,300
Incubation time of mAb	0,261	−0,243
Incubation time of FITC-conjugated	0,239	0,4
Volume of mAb	0,415	0,457
Volume of FITC-conjugated	0,319	−0,301
Formaldehyde cell fixation	**0,695^a^**	**1,333^a,b^**
Dummy Factor	0,125	−0,152
**Mean of modular values of effects**	**0,261**	**0,245**
**Experimental error estimated according algorithm of Dong**		
s_0_	0,3915	0,3675
2,5× s_0_	0,97875	0,91875
s_1_	0,36477	0,26894
ME (α=0,05)	**0,803**	**0,599**
ME (α=0,1)	**0,655**	**0,487**

a Significant at α=0,1;

b Significant at α=0,05; s_0_= Estimate of error; s_1_=Standard errror; ME =margin of error.

**Tab. 3.** Specificity data for nimotuzumab binding assay.

**Samples**		**% of Binding (Average)**		**Acceptance criteria (%)**
		**A 431**	**NCI-H125**	
Isotype mAb	T1hT	3,07	0,87	< 5,00
Matrix	Formulation solution	1,97	0,50	< 5,00
	Culture supernatant	1,37	0,83	< 5,00
Nimotuzumab	Reference standard	99,93	99,97	≥97,00
	Culture supernatant	99,97	100	≥97,00
	Degraded sample	92,77	67,93	< 97,00

**Tab. 4.** Precision results. Average, Standard Deviation and RSD of six replicates over 3 days.

**Cell Line**			**A 431**			**NCI-H125**		

			**Average**	**SD**	**RSD**	**Average**	**SD**	**RSD**
**Intra assay**								

Analyst 1	Day 1	FP	99,68	0,16	0,16	99,85	0,08	0,08
		CS	99,48	0,30	0,30	99,83	0,23	0,23
	Day 2	FP	99,40	0,39	0,40	99,58	0,08	0,08
		CS	99,70	0,17	0,17	99,78	0,12	0,12
	Day 3	FP	99,25	0,38	0,38	99,52	0,29	0,29
		CS	99,65	0,10	0,11	99,53	0,47	0,47

Analyst 2	Day 1	FP	98,62	0,84	0,85	99,28	1,03	1,04
		CS	99,43	0,25	0,25	99,15	1,41	1,43
	Day 2	FP	98,30	0,72	0,73	99,97	0,05	0,05
		CS	99,65	0,19	0,19	99,95	0,08	0,08
	Day 3	FP	99,45	0,39	0,40	99,23	0,82	0,83
		CS	99,77	0,19	0,19	98,85	1,64	1,66

**Inter assay**								

Analyst 1		FP	99,44	0,36	0,36	99,65	0,22	0,22
		CS	99,61	0,22	0,22	99,73	0,33	0,33
Analyst 2		FP	98,79	0,81	0,82	99,49	0,79	0,80
		CS	99,62	0,24	0,24	99,32	1,27	1,28

Inter analyst								

2 Analysts		FP	99,12	0,70	0,71	99,57	0,58	0,58
		CS	99,61	0,23	0,23	99,53	0,94	0,94

Data is shown as % of binding (Average, SD and RSD). Nimotuzumab is evaluated as purified final product (FP) and culture supernatant (CS).

**Tab. 5.** Factors and levels evaluated in the robustness assays.

**Factor**	**ID**	**Factor classific.**	**Limit**	**Low Level (−)**	**High Level (+)**	**Nominal Level**
Cell Culture Media manufacturer	**G**	qual.	–	GIBCO	PAA	GIBCO
Fetal Bovine Sera manufacturer	**E**	qual.	–	GIBCO	PAA	GIBCO
Time of Trypsinization	**I**	quant.	±1 min	11 min (A431) 2 min (H125)	13 min (A431) 4 min (H125)	12 min (A431) 3 min (H125)
Trypsin-EDTA manufacturer	**B**	qual.	–	GIBCO	PAA	GIBCO
Incubation time for EGFR recovery	**A**	quant.	±10 min	50 min	70 min	60 min
Incubation time of mAb	**C**	quant.	±5 min	25 min	35 min	30 min
Incubation time of FITC-conjugated	**H**	quant.	±5 min	25 min	35 min	30 min
Volume of mAb	**D**	quant.	±5 μL	15 μL	25 μL	20 μL
Volume of FITC-conjugated	**K**	quant.	±5 μL	15 μL	25 μL	20 μL
Formaldehyde cell fixation	**J**	qual.	–	No	Yes	No
